# Exercise-related leg muscle signal changes: assessment using diffusion-weighted MRI

**DOI:** 10.1186/s41747-023-00323-2

**Published:** 2023-03-08

**Authors:** Floriane Kolmer, Guillaume Bierry, Thibault Willaume

**Affiliations:** MSK Imaging, University Hospital of Strasbourg, 1 Avenue Molière, 67098 Strasbourg, France

**Keywords:** Adult, Diffusion magnetic resonance imaging, Leg, Healthy volunteers, Muscles

## Abstract

We aimed to detect physiologic changes of leg muscle signal on magnetic resonance (MR) diffusion-weighted imaging (DWI) in asymptomatic subjects after repetitive plantar flexion exercises. In this monocentric prospective study, DWI of both legs were performed at rest and after exercise periods (5 min, Ex_5_: and 10 min, Ex_10_) in 20 active healthy subjects (mean age 31 years). The exercise consisted in repetitive plantar flexion of the right foot using elastic band, the patient being sited directly on the MR table. Both visual semiquantitative evaluations and quantitative (apparent diffusion coefficient, ADC; fractional anisotropy, FA) were performed in 5 leg compartments. Visually, signal changes involved mainly the fibular and gastrocnemius muscles and were considered intense after Ex_5_ in 3 subjects, moderate only after Ex_5_ in 10, and moderate only after Ex_10_ in 4. No changes were visible in 3 subjects. Quantitative evaluation confirmed significant signal changes in the fibular (ADC: + 17.4%, *p* < 0.001; FA: -8.3%, *p* = 0.030) and gastrocnemius (ADC: + 13.7%, *p* < 0.001; FA: -11.4%, *p* < 0.001) muscles between rest and post-exercise MR. Plantar flexion exercises induce changes on DWI, especially in fibular and gastrocnemius muscles, which can be visually and quantitatively detectable in asymptomatic active subjects.

**Trial registration**

EudraCT 2008-A00694-51.

**Supplementary Information:**

The online version contains supplementary material available at 10.1186/s41747-023-00323-2.

## Key points

- Physiologic changes of muscles magnetic resonance (MR) signal exist immediately after exercise.
- Changes can be observed visually and quantitatively on MR diffusion-weighted imaging.
- Such changes might reflect normal muscle adaptation to exercise.

## Background

Magnetic resonance (MR) diffusion-weighted imaging (DWI) is a promising tool for the exploration of post-exercise muscle changes, due to its high sensibility to water change within tissue [1]. Nevertheless, to be used in routine, several technical limitations of MR have to be solved. Standardised protocols have to be proposed, and a deeper knowledge of the normal muscle appearance on MR during exercise is required.

Indeed, muscle MR changes can be subtle and versatile and might have disappeared by the time the patient is repositioned within the MR system and re-scanned. Thus, to obtain reproducible and comparable results, the protocol (MR sequence, patient installation, exercise setup) should be simple, short, and sensitive at the same time. In addition, the normal behaviour of muscles signal during exercise is, to date, vastly underinvestigated, and the knowledge of potential physiologic changes is a requisite for a reliable interpretation [2].

In this study, we aimed to propose a simple, standardised MR protocol that allows the exploration of calf muscles immediately after exercise and report normal DWI changes in asymptomatic active subjects. We indeed hypothesise that exercise-related physiological changes can be detected using MR DWI.

## Methods

### Subjects

Twenty healthy subjects were prospectively recruited from the medical personnel of our institution from May 2021 to September 2021. Subjects must have been free of any musculoskeletal disorders that might have affected leg muscles physiology. A self-assessment physical activity questionnaire was used to assess the level of physical activity of the patients included [3]. As we had none a priori knowledge, a preliminary study was performed on five of those subjects using the MR setup described below. As signal changes were already observed in four of them, we arbitrarily chose to include a total of 20 subjects.

### Exercise and MR setup

First, the subjects were placed on the MR unit table in the supine position, and MR of both legs was performed at rest. Then, without getting off the examination table, the subjects were placed in a seated position with their legs extended. They were asked to perform a repeated concentric plantar flexion of the right foot against the resistance of a commercially available elastic band. Subjects were asked to hold the band with their hands and achieve maximum plantar flexion for each movement with a pace of 50 to 60 repetitions per min (Supplementary video). The protocol was then as follows: rest MR (MR_1_); 5-min exercise (Ex_5_); second MR (MR_2_); 5-min rest; 10-min exercise (Ex_10_); third MR (MR_3_). MR_2_ and MR_3_ were performed immediately at completion of the exercise.

### MR sequences

All studies were performed on a 3-T MR unit (Ingenia Elition X, Philips, Best, The Netherlands) with a 32-channel torso coil that allowed a simultaneous bilateral acquisition. The protocol consisted in an unique fat-suppressed echo planar DWI sequence using the following parameters: *b* = 800 s/mm^2^; directions = 12; repetition time = 3,150 ms; echo time = 80 ms; matrix = 116 × 85; field of view = 350 × 252 mm; slice thickness = 5 mm; number of slices = 36; acquisition time = 2 min 50 s. Apparent diffusion coefficient (ADC) and fractional anisotropy (FA) maps were automatically generated by the MR unit.

### Image evaluation

Both visual semiquantitative and quantitative evaluations of muscle signal were performed in five different compartments: anterior (tibialis anterior, extensor hallucis longus, extensor digitorum longus), lateral (peroneus longus and brevis), deep posterior (tibialis posterior, flexor hallucis longus and flexor digitorum longus), intermediate posterior (soleus), and superficial posterior (medial and lateral gastrocnemius).

**Fig. 1:**
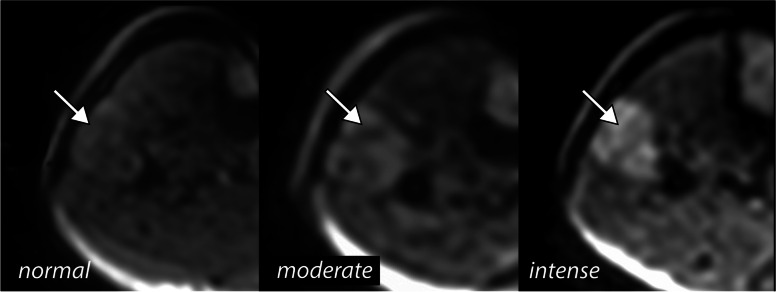
Visual scale of signal change at the level of the lateral compartment (arrows)

The visual semiquantitative evaluation was performed separately by two readers (F.K., R1, a 4-year resident; T.W., R2, a senior radiologist with 5 years of experience in musculoskeletal imaging) on the diffusion-weighted trace images. A three-grade scale was used: 0 = no signal changes, 1 = moderate signal changes, 2 = intense signal changes (Fig. 1). The contralateral leg served as a reference for evaluation. In cases of discrepancies between the two readers, consensus was obtained by a third reader (G.B., R3, a senior radiologist with 15 years of experience in musculoskeletal imaging).

The quantitative evaluation was performed on the ADC and FA maps separately by two readers (R1, R2) using 150 mm^2^ regions of interest. Measures were performed on corresponding levels (10 cm distal to the tibial plateau) and repeated twice by each reader after a 10-day interval; the mean values of the 4 measures were kept for further analysis. Because of its size, the signal of the superficial posterior compartment was measured at the medial gastrocnemius level.

## Statistical analysis

Analysis were performed using the SPSS software (v 24.0, IBM, Chicago, USA). Changes of ADC and FA values were compared using ANOVA for repeated measures with post hoc Bonferroni tests. Interobserver agreements were calculated for ADC and FA using two-way random intraclass coefficient based on R1 and R2 first evaluation and for visual evaluation using unweighted Cohen’s *κ*. Agreements were considered slight (0.00–0.20), fair (0.21–0.40), moderate (0.41–0.60), substantial (0.61–0.80), and almost perfect (0.81–1.00) [4]. A *p* < 0.05 was retained for statistical significance.

## Results

### Population

Twenty healthy subjects consisting of 10 males of a mean age 28 years (range 24–32) and 10 females of a mean age of 34 (range 23–50) were included. The physical activity profile was as follows: inactive (no subjects), active (20 subjects), and very active (no subjects).

### Semiquantitative visual evaluation

**Fig. 2:**
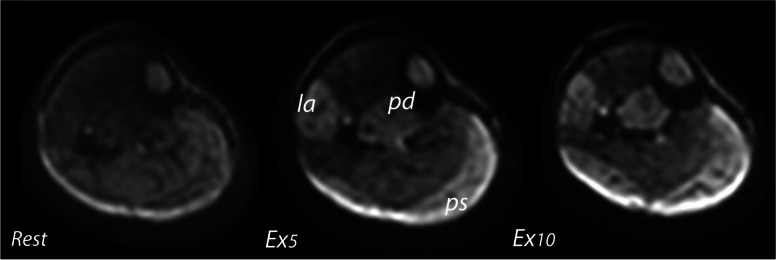
Axial diffusion-weighted TRACE images in a 22-year-old subject. Intense changes were seen in the lateral (*la*), posterior deep (*pd*), and posterior superficial (*ps*) after both the 5-min (Ex_5_) and the 10-min exercises (Ex_10_)

Signal changes were visually apparent in 17 of the 20 subjects (85% of all subjects): intense (grade 2) at both Ex_5_ and Ex_10_ in 3 subjects, moderate (grade 1) at both Ex_5_ and Ex_10_ in 10 subjects, moderate (grade 1) only at Ex_10_ in 4 subjects (Table 1) (Fig. 2). Three subjects did not present any visual changes of muscle signal, neither after Ex_5_ nor Ex_10_. Changes were observed in the lateral (15 of 20 subjects, 75%), posterior superficial (9 of 20 subjects, 45%), and posterior deep compartment (2 of 20 subjects, 10%).

**Table 1:** Changes of ADC values (μm^2^/ms) and FA in muscle compartments on quantitative evaluation after each exercise

Parameter, compartment	Rest		Ex_5_			Ex_10_			
	Mean	SD	Mean	SD	% change^a^	Mean	SD	% change^a^	% change^b^
ADC, anterior	1.53	0.09	1.59	0.10	3.9	1.59	0.15	3.9	0
ADC, lateral	1.61	0.09	1.76	0.14	9.3^#^	1.89	0.18	17.4^#^	7.4^#^
ADC, posterior deep	1.46	0.14	1.60	0.15	9.6	1.62	0.17	10.9	1.2
ADC, posterior intermediate	1.55	0.08	1.57	0.07	1.3	1.57	0.06	1.3	0
ADC, posterior superior	1.46	0.14	1.61	0.15	10.3^#^	1.66	0.17	13.7^#^	3.1
FA, anterior	0.38	0.03	0.38	0.03	0	0.39	0.05	2.6	2.6
FA, lateral	0.36	0.03	0.35	0.03	-2.8^#^	0.33	0.02	-8.3^#^	-5.7^#^
FA, posterior deep	0.37	0.04	0.38	0.04	2.7	0.39	0.03	5.4	2.6
FA, posterior intermediate	0.35	0.03	0.36	0.02	2.8	0.35	0.02	0	-2.8
FA, posterior superior	0.35	0.03	0.33	0.02	-5.7^#^	0.31	0.02	-11.4^#^	-6.1^#^

*ADC* Apparent diffusion coefficient, *FA* Fractional anisotropy, *SD* Standard deviation

^a^% of changes relative to rest

^b^% of changes relative to Ex_5_

^#^Statistically significant changes (*p* < 0.05)

### Quantitative evaluation

Inter-reader agreement (intraclass correlation coefficient) were 0.77 (95% confidence interval 0.32–0.92) for ADC evaluation and 0.82 (confidence interval 045–0.94) for FA evaluation. For visual evaluation, Cohen’s *κ* was 0.78 (95% confidence interval 0.68–.087). Statistically significant changes in ADC and FA after exercise were seen in two compartments, the lateral and posterior superficial ones (Table 1). In the lateral compartment, ADC increased between rest and Ex_5_ (+ 9.3%; *p* = 0.004), between Ex_5_ and Ex_10_ (+ 7.4%; *p* = 0.020), and between rest and Ex_10_ (+ 17.4%; *p* < 0.001). FA decreased between rest and Ex_5_ (-2.8%; *p* = 0.010), between Ex_5_ and Ex_10_ (-5.7%; *p* = 0.020), and between rest and Ex_10_ (-8.3%; *p* = 0.030).

In the posterior superficial compartment, ADC significantly increased between rest and Ex_5_ (+ 10.3%; *p* = 0.001) and between rest and Ex_10_ (13.7%; *p* = 0.001), but not between Ex_5 and_ Ex_10_ (*p* = 0.120). FA decreased between rest and Ex_5_ (-5.7%; *p* = 0.030), between Ex_5_ and Ex_10_ (-6.1%; *p* = 0.040), and between rest and Ex_10_ (-11.4%; *p* < 0.001).

## Discussion

Due to its high sensitivity to water content changes that accompanies muscle suffering, MR is certainly an interesting tool for the noninvasive investigation of post-exercise muscle change, but its precise role remains to be defined [5]. Indeed, to date, the exploration of exercise-induced muscle signal changes by MR remains challenging because of two main limitations: first, MR changes can be subtle and only transitory, and second, whether those variations might be physiologic adaptations rather than true muscle injuries remains incompletely explained [1].

To overcome those limitations, we presented a simple standardised protocol MR setup allowing to perform immediate post exercise scanning. Subjects were asked to perform foot flexion against resistance while still on the MR table using commercially available elastic bands. This was done to limit the time interval between exercise and MR examination, as the MR sequence could be started immediately at the end of the effort.

We used a DWI sequence because of its high sensitivity to changes in water content and ability to reveal early and subtle muscle oedema [6, 7]. If DWI has been widely investigated in nerve-related (neurogenic) or inflammatory and tumour muscle disorders, there are few reports about normal muscle behaviour during exercise [8–11].

We observed that in active patients, exercise induces an increase in water content in muscles that can be detected both visually and quantitatively using DWI. In addition to changes in ADC values, we observed statistically significant decreases of FA in several muscle compartments, presumably resulting from a modification of muscle well-organised architecture during exercise [12]. The changes likely reflect the normal adaptation of the muscles during exercise, with an increase in water, hyperaemia, and vasodilatation regardless of vascular patency [13, 14].

Our study presented several limitations. First, we only included a relatively small group of 20 subjects. However, we did our best to have, as much as possible, a homogenous population of young and active subjects. Second, the amplitude of flexion and, therefore, the level of muscle activation performed by each subject might be variable. Last, Ex_5_ and Ex_10_ were performed consecutively and it is not certain that the muscle signal was back to normal during this interval.

To summarise, DWI was able to demonstrate exercise-induced changes within leg muscles in asymptomatic patients, and such changes are presumably the normal response of the muscles to their activation.

## Supplementary Information

**Additional file 1:Supplementary video.** exercise and MR setup.

## Data Availability

The datasets used and/or analysed during the current study are available from the corresponding author on reasonable request.

## Abbreviations

ADC: Apparent diffusion coefficient
FA: Fractional anisotropy
MR: Magnetic resonance
